# Chitin Extracted from the Shell of Blue Swimming Crabs (*Portunus pelagicus* Linn.) Inhibits NF-kappaB p65 in Ethanol-Induced Gastric Ulcerative Wistar Rats

**DOI:** 10.3390/md21090488

**Published:** 2023-09-12

**Authors:** Renny Amelia, Sri Adi Sumiwi, Nyi Mekar Saptarini, Jutti Levita

**Affiliations:** 1 Department of Pharmacology, School of Pharmacy Muhammadiyah Cirebon, Cirebon 45153, West Java, Indonesia; 2 Department of Pharmacology and Clinical Pharmacy, Padjadjaran University, Sumedang 45363, West Java, Indonesia; sri.adi@unpad.ac.id; 3 Department of Pharmaceutical Analysis and Medicinal Chemistry, Padjadjaran University, Sumedang 45363, West Java, Indonesia; nyi.mekar@unpad.ac.id

**Keywords:** cytokines, gastric ulcer, inflammatory, N-acetylglucosamine, NF-kappaB p65

## Abstract

Peptic ulcer disease is generated by the activation of NF-kappaB activity. A recent clinical study reported a significant increase in NF-kappaB2 gene expression in 79 samples of peptic ulcer patients compared to the control group. Moreover, the deacetylated chitin could alter the translocation of NF-kappaB p65 to the nucleus. Considering this, our work aims to explore the effect of chitin extracted from the shell of blue swimming crabs (*Portunus pelagicus* Linn.) towards NF-kappaB p65 levels in ethanol-induced gastric ulcerative Wistar rats. The shells are found abundantly as the waste of seafood processing in the northern part of West Java, Indonesia. In this study, chitin extraction was carried out using the microwave-assisted extraction method by employing choline chloride (C_5_H_14_ClNO) and DL-malic acid (C₄H₆O₅) as the solvents. The inhibitory activity assay of chitin on the expression of NF-kappaB p65 was performed by using Western blot. The extraction yielded a good quality of chitin with a deacetylation degree of 30.8026%, molecular weight of 3.35 × 10^5^ Da, and a negligible heavy metals level. Moreover, chitin extract at doses of 150, 300, and 600 mg/kg BW significantly reduced the percentage of gastric ulcer index compared to the negative control group. Meanwhile, chitin extract at doses of 300 and 600 mg/kg BW significantly inhibited NF-kappaB expression compared to the negative control group. Histopathological examination demonstrated a decrease in the number of necrotic cells and fat degeneration in the gastric mucosa and an increase in normal cells. Taken together, chitin extract obtained from the shells of blue swimming crabs may be able to prevent gastric ulcers induced by ethanol via the inhibition of NF-kappaB p65; however, further studies are needed to verify its anti-ulcerative properties.

## 1. Introduction

The activation of nuclear factor-kappaB (NF-kappaB) is governed by various mechanisms, e.g., interactions with its inhibitors. In the cell cytoplasm, NF-kappaB occurs in an inactive form as a complex with its inhibitor, IkappaB. Due to its capacity to stimulate the expression of various genes, the activity of NF-kappaB is firmly controlled at numerous stages [1,2,3]. NF-kappaB plays an important role as a mediator in the immune system. It contributes to stress responses and regulates cell proliferation and programmed cell death [3]. NF-kappaB is activated by proinflammatory factors such as interleukins and by *Helicobacter pylori* infection during the development of peptic ulcers. The NF-kappaB stimulation occurs when the IkappaB kinase complex is phosphorylated and eventually leads to the degradation of IkappaB. Inhibiting NF-kappaB leads to protection against long-term intestinal inflammation in animal models [4]. NF-kappaB was reported to be present in its active form in the healing phase of rats with gastric ulcers. Inflammatory cytokines, such as interleukin-1β, cyclooxygenase-2, and inducible nitric oxide synthase contribute to the development of gastric ulcers. Preventing the activation of NF-kappaB led to the recovery of gastric ulcers in rats [5]. Interestingly, a recent study in humans reported a significant increase in NF-kappaB2 gene expression in seventy-nine samples taken from patients diagnosed with peptic ulcers. However, no notable distinctions occurred in the expression of the NFKB2 gene between the patients infected and uninfected with *H. pylori* [6].

Chitin is a long-chain polymer of N-acetylglucosamine with β-(1–4) linkages, contained in the shell of crustaceans. It prevails in three isoforms, α-, β-, and γ-structures. The α-structure exists bountifully in nature [7]. A previous study reported that β-chitin had been successfully extracted from the exoskeleton of *Portunus pelagicus* Linn. [8]. During extraction, the shells of Crustaceans are reacted with an inorganic acid to dissolve calcium carbonate, followed by the addition of a strong base to remove protein [9].

Chitin has been reported for its activity in suppressing the production of proinflammatory interleukin-8 [7]. Different sizes of chitin also regulate the production of macrophage tumor necrosis factor (TNF) and IL-10 in C57BL/6 mice [10]. Chitin can upregulate and downregulate the effect of the NLR family pyrin domain containing 3 (NLRP3) inflammasomes based on its preparation [11]. Deacetylated chitin altered the translocation of NF-kappaB p65 to the nucleus as proven by the significant decrease of the mRNA expression of p65 under heat stress compared to the negative control group (*p* < 0.05) [12]. Chitin extracted from shrimp and squilla shell waste was reported to exhibit antioxidant activity and inhibit the growth of *Staphylococcus aureus* and *Escherichia coli* [13].

In the northern part of West Java, Indonesia, blue swimming crab shells are present lavishly as waste from the processing of seafood. Up to a point, these shells have not been utilized for their health and economic values; accordingly, our work aims to explore whether chitin contained in these shells possesses an anti-inflammatory activity via the inhibition of NF-kappaB p65 in ethanol-induced gastric ulcerative Wistar rats. 

## 2. Results

### 2.1. The Taxonomy Identification of the Crab Shells and the Physicochemical Properties of the Crab Shell Powder and the Chitin Extract

**Figure 1:**
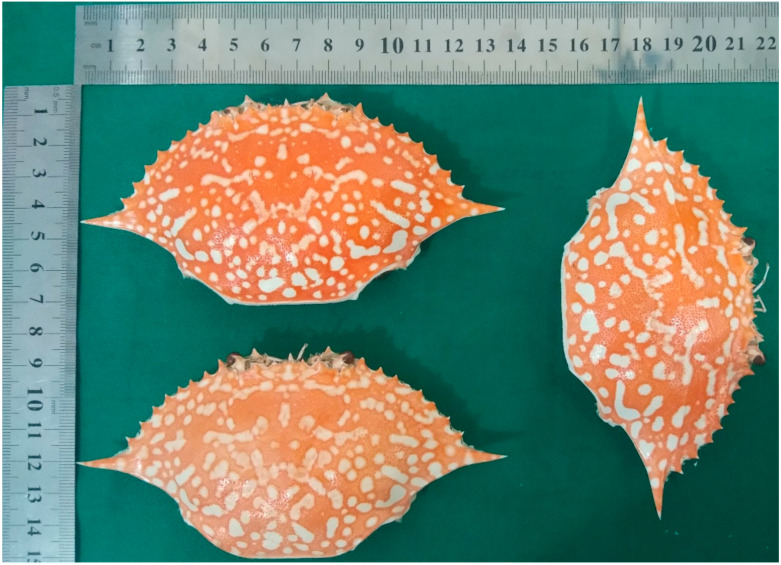
The after-boiled shells of blue swimming crabs (*Portunus pelagicus* Linn.) were collected in Gunung Jati Cirebon, West Java, Indonesia.

The fresh blue swimming crab shells (Figure 1) were identified at the Zoology Museum-Laboratory of Identification and Determination, School of Life Sciences and Technology, Bandung Institute of Technology, and based on characteristics described by Poore (2004) [14], Lai et al. (2010) [15], were confirmed as *Portunus pelagicus* Linn. (No. 2974/11.C02.2/2019).

The physicochemical properties of the crab shell powder and the chitin extract, respectively, were as follows: the water content was 4.53% and 0.88%, protein was 8.17% and 6.30%, lipid was 0.26% and 0.28%, and carbohydrate was 15.73% and 55.86.

The levels of five toxic heavy metals of the crab shell powder and the chitin extract, respectively, were as follows: mercury was 5.73 ± 0.005 mg/kg and <0.0001 mg/kg, lead was 3.46 ± 0.24 and <0.0001 mg/kg, chromium was 2.55 ± 0.00 mg/kg and 13.09 ± 0.02 mg/kg, cadmium was 0.29 ± 0.00 mg/kg and <0.0001 mg/kg, and arsenic was 64.16 ± 2.67 mg/kg and <0.0001 mg/kg.

**Figure 2:**
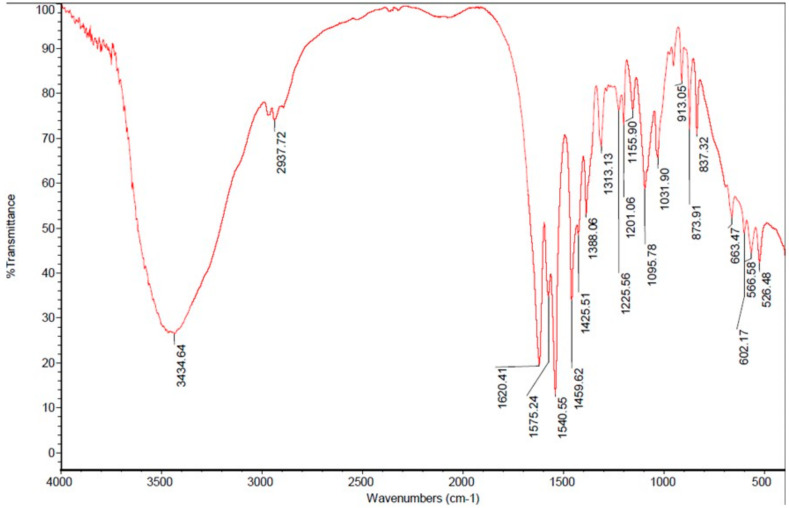
The % transmittance FT-IR spectrum of chitin extracted from the shells of blue swimming crabs (*P. pelagicus* Linn.) recorded at 4000 to 500 cm^−1^ revealing the OH stretch band at 3434.64 cm^−1^ superimposed on NH stretching band (occurs as shoulder bands at 2937.72 cm^−1^); the amide C=O band at 1620.41 cm^−1^; the amide –CN band at 1425.51 cm^−1^; the alkyl C-H band at 1388.06 cm^−1^; the −CN of amino groups between 1155.90 and 1201.06 cm^−1^; and the polysaccharide bands are detected between 566.58 and 1031.90 cm^−1^.

FT-IR spectrum of chitin extract (depicted in Figure 2) demonstrated the amide C=O band at 1620.41 cm^−1^ (%T value of 19.805% or A = −1.2967) and the OH stretch band at 3434.64 cm^−1^ (%T value of 26.441% or A = −1.4222). Thus, the deacetylation degree (DD) of chitin extract, determined by calculating the absorbance of these bands, was 30.8026%. 

The intrinsic viscosity (***η***) of chitin 1% solution in acetic acid at 27.3 °C was 1.9750 ± 0.031 centipoise (cP). By using the Mark–Houwink equation, the molecular weight of chitin was obtained at 3.35 × 10^5^ Da.

### 2.2. Effect of Chitin Extract, Commercial Chitin, and Shell Powder on the % Relative Organ Weight and % Ulcer Index

**Figure 3:**
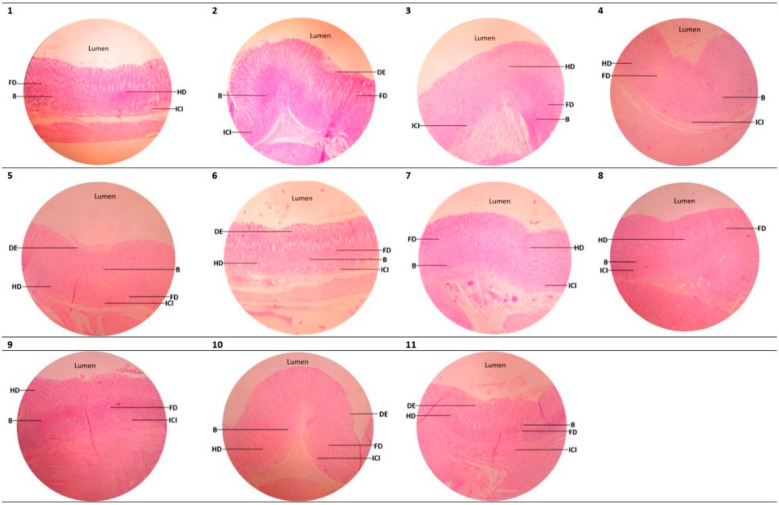
Hematoxylin-eosin staining of rat stomach organs (B = bleeding, DE = desquamated epithelium, FD = fat degeneration, HD = hydropic degeneration, ICI = inflammatory cell infiltration: (1) positive control group; (2) negative control group; (3) normal control group; (4–6) chitin extracts (150 mg/kg BW, 300 mg/kg BW, and 600 mg/kg BW); (7–9) commercial chitins (150 mg/kg BW, 300 mg/kg BW, and 600 mg/kg BW); (10, 11) crude shell powder (500 mg/kg BW and 1000 mg/kg BW). All microscopic images used an ocular lens magnification of 12× and an object lens of 10×. All experiments were replicated 3×.

The results of hematoxylin-eosin (HE) staining are depicted in Figure 3.

All doses of chitin extract, commercial chitin, and crude shell powder demonstrate significant differences in % ulcer index compared to the negative control group. The effect exhibited by chitin extract, commercial chitin, and crude shell powder is similar to the rats treated with sucralfate (the positive control group) (Table 1). There is no significant difference in the % relative organ weight of all treated rat groups compared to the negative control group (Table 1).

### 2.3. Effect of Different Doses of Chitin Extract, Commercial Chitin, and Shell Powder on the Hemorrhagic Severity of the Gastric Mucosa in Ethanol-Induced Gastric Ulceration Rats

**Figure 4:**
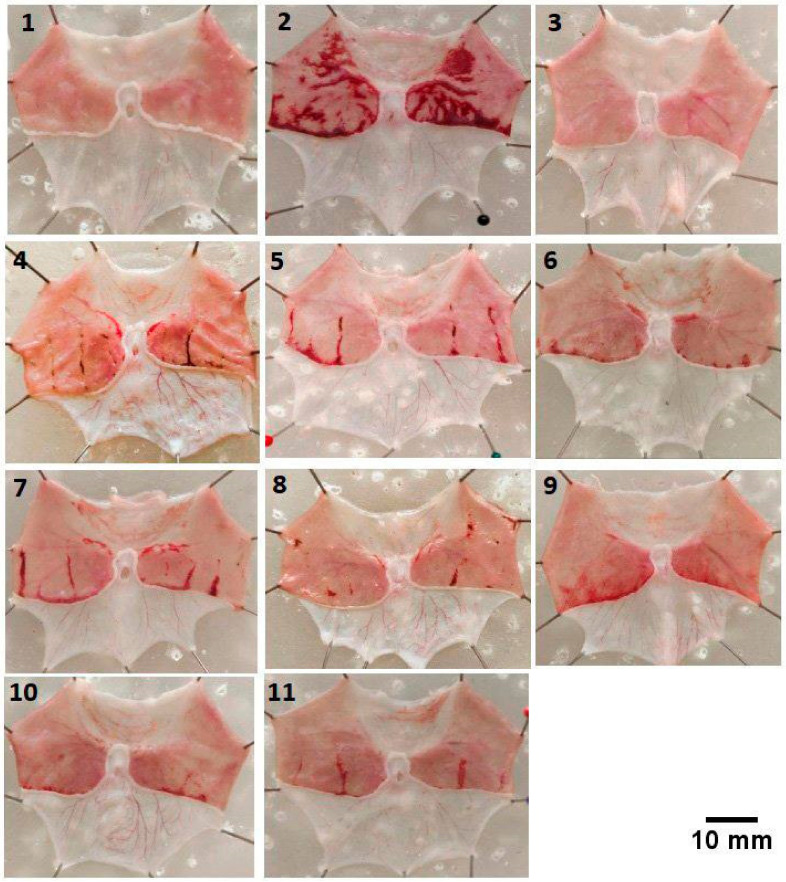
Effect of different doses of chitins in the form of extracts, commercials, and shell powders on the hemorrhagic severity of the gastric mucosa in ethanol-induced gastric ulceration rats: (1) positive control group: intact gastric mucosa tissues; (2) negative control group: severe hemorrhage; (3) normal control group; (4–6) chitin extracts (150 mg/kg BW, 300 mg/kg BW, and 600 mg/kg BW): mild hemorrhagic on the gastric mucosa; (7–9) commercial chitins (150 mg/kg BW, 300 mg/kg BW, and 600 mg/kg BW): mild hemorrhagic on the gastric mucosa; (10, 11) shell powders (500 mg/kg BW and 1000 mg/kg BW): mild hemorrhagic on the gastric mucosa. All experiments were replicated 3×.

Inducing rats with ethanol (5 mL/kg BW, orally by intragastric gavage) caused a severe gastric lesion (Figure 4 and Table 2). The pretreatment with sucralfate, the control drug (1080 mg/kg BW, orally), could protect the stomach against ethanol-induced gastric mucosa hemorrhage. Similarly, higher doses of chitins in the form of extracts, commercials, and crude shell powders could reduce the severity of ethanol-induced damage (Figure 4). Nonetheless, pretreatment using lower concentrations of chitin extract (150 mg/kg BW) resulted in only weak protection.

### 2.4. Effect of Different Doses of Chitin Extract, Commercial Chitin, and Shell Powder on the Expression of NF-kappaB p65 in Western Blot Analysis

**Figure 5:**
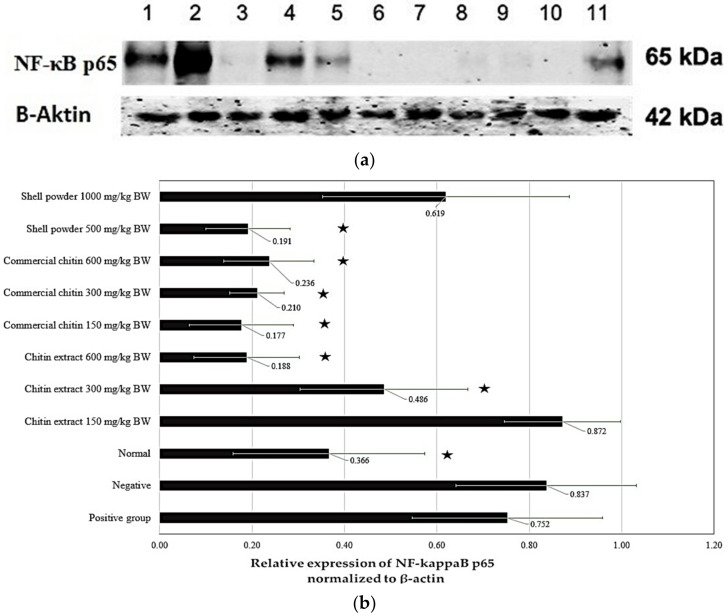
(**a**) The bands of NF-kappaB p65 (65 kDa) and β-actin (42 kDa) in the stomach of the Wistar rats by Western blot analysis; (**b**) The relative expression of NF-kappaB p65 normalized to β-actin in all rat models: (1) positive control group; (2) negative control group; (3) normal control group; (4–6) chitin extracts (150 mg/kg BW, 300 mg/kg BW, and 600 mg/kg BW); (7–9) commercial chitins (150 mg/kg BW, 300 mg/kg BW, and 600 mg/kg BW); (10, 11) shell powders (500 mg/kg BW and 1000 mg/kg BW). * *p* < 0.05 means significantly different compared with the negative control group. All experiments were replicated 3×.

Figure 5 shows the bands of NF-kappaB p65 (molecular weight of 65 kDa) and β-actin (molecular weight of 42 kDa) in the stomach of the Wistar rats, respectively. Chitin extracts (dose of 300 mg/kg BW and 600 mg/kg BW), all doses of commercial chitin, and crude shell powder dose of 500 mg/kg BW could significantly reduce the expression of NF-kappaB p65 in the stomach of ethanol-induced gastric ulcerative rats. The inhibitory activity on NF-kappaB p65 expression by chitin extracts is better than that of sucralfate (the positive control drug). However, an increase in the relative expression of NF-kappaB p65 was observed at the lowest dose of chitin extract (150 mg/kg BW) and the highest dose of crude shell powder (1000 mg/kg BW).

## 3. Discussion

The main findings of our work are (1) chitin extracted from the shells of blue swimming crabs at doses of 150, 300, and 600 mg/kg BW significantly reduced the percentage of gastric ulcer index in ethanol-induced gastric ulcerative rats; (2) chitin extract at doses of 300 and 600 mg/kg BW significantly inhibited NF-kappaB levels compared to the negative control group.

It was confirmed that excessive alcohol drinking had demonstrated damage to the gastrointestinal tract. A previous prospective study on 48,000 males aged 40–75 years reported that those who consumed >30 g/day of alcohol exhibited a notable risk for major gastric bleeding when compared to non-alcohol drinkers [16]. Studies on the endoscopic examination of thousands of patients with peptic ulcers confirmed that there was a correlation between this disease and the risk factors, among which is alcohol consumption [17,18]. These studies have been the reason why ethanol was chosen for the gastric-ulcerative inducer in our study.

Based on its source, chitin in nature presents in two structures, α-chitin, which is the most abundant, and β-chitin [19]. Numerous studies reported the source of chitin extraction, e.g., from the exoskeletons of prawns [20], shrimps [21,22,23,24], and crabs [8,12,25,26].

In the present study, chitin extracted from the shells of blue swimming crabs was assayed for its water (0.88%), protein (6.30%), lipid (0.28%), and carbohydrate (55.86) contents. Chitin extract was confirmed safe as proven by the low levels of five toxic heavy metals (mercury < 0.0001 mg/kg, lead < 0.0001 mg/kg, chromium 13.09 ± 0.02 mg/kg, cadmium < 0.0001 mg/kg, and arsenic < 0.0001 mg/kg). The deacetylation degree (DD) of the extract was 30.8026% and the molecular weight was 3.35 × 10^5^ Da, which further proved the presence of chitin and the quality of the extract.

A deacetylation degree (DD) value of <60% indicates the presence of chitin, whereas a DD of >60% belongs to chitosan [27,28]. It was reported that the DD value of chitin obtained from mud crab shells collected in Brunei Darussalam was 48.0% [29]. Another FTIR analysis on chitin extracted from *Labeo rohita* fish collected in Rourkela, India revealed a DD value of 61% [30]. Moreover, the DD value of chitin extracted from the shrimp waste collected in a local fish market of Buraidah, Qassim, Kingdom of Saudi Arabia was 70.9% at pH 8.4 [31].

Meanwhile, the present study revealed that pretreatment with chitin could protect the gastric mucosal cells against ethanol-induced ulcerative damage. It seems that the gastroprotective activity of chitin also advances the immune system. A previously reported clinical trial on 24 volunteers confirmed chitin activates the immune system [32], by inducing the production of type 1 cytokines, thus preventing type 2 inflammation [33]. It was reported chitin could activate T cells and natural killer cells, and stimulate the synthesis of interferon-γ [34].

In our study, chitin extracts could significantly reduce the expression of NF-kappaB p65 in the stomach of ethanol-induced gastric ulcerative rats. Although there is a very limited report on the inhibitory activity of chitin against NF-kappaB, a recent study confirmed that the deacetylated form of chitin in nanoparticles significantly reduced mRNA gene expression of GSK-3, TNF-α, and NF-kappaB. NF-kappaB is activated through oxidative stress, which eventually induces an inflammatory cascade by initiating the gene transcription of many proinflammatory cytokines [35].

Moreover, the ulcer healing process requires several biological and molecular reactions, including inflammatory response and cell migration. Therapeutic strategy for inflammatory diseases aims to block NF-kappaB activity in several mechanisms, (1) by inhibiting IKK activity; (2) by suppressing protease activity; (3) by inhibiting the translocation of NF-kappaB to the nucleus; thus, the NF-kappaB subunits such as RelA (p65), p50, c-Rel, and other NF-kappaB subunits will be prevented from entering the nucleus; and (4) by preventing the binding of NF-kappaB subunits with DNA [36]. However, a dose of 300 mg/kg BW of purified chitosan, the deacetylated derivative of chitin, demonstrated an anti-inflammatory activity on mice infected with enterotoxigenic *Escherichia coli* [37]. A dose of 16 mg/kg BW of purified chitin oligosaccharide was reported to reduce inflammation on ovalbumin (OVA)-induced lung inflammation in a mouse model of asthma [38]. A dose of 10 mg/kg BW of pure chitosan (purity 98%) decreased the AST, ALT, and ALP, and improved the histology of CCl4-induced hepatic fibrosis (HF) rat models [39].

Our study confirms that chitin alleviates peptic ulcers by inhibiting the translocation of the NF-kappaB p65 subunit to the nucleus, as shown by the disappearance of the NF-kappaB p65 bands in the Western blot electropherogram (Figure 5). Similarly, it was reported in a previous study that chitin extracted from cuttlebone was able to heal wound lesions in rat models. Cuttlebone chitin can stimulate macrophages and increase the expression of TNF-α, IL-1β, and IL-6. Increased production of IL-8 also occurs due to fibroblast migration, thereby accelerating the wound-healing process [7].

Chitin activates cytokine production, leukocyte recruitment, and macrophage activation. It is sensed by the immune system via specific membrane-bound receptors and is important in defense against pathogens [40].

However, the limitation of our study is lacking the data on IL-6 expression. IL-6 is a biomarker for gastric inflammation [41], thus opening the chance for future studies that an evaluation of another NF-kappaB such as IL-6, GSK-3, and TNF-α can be carried out.

## 4. Materials and Methods

### 4.1. Chemicals and Antibodies

Choline chloride (Salus Nutra Inc., Xian, China), DL-malic acid (Salus Nutra Inc., Xian, China), commercial chitin (Carbosynth, China), sucralfate (Combiphar, Bandung, Indonesia), ethanol, pure 99.5% (Merck Millipore-Sigma Aldrich, USA), ethyl ether (Merck Millipore-Sigma Aldrich, St. Louis, MI, USA), carboxymethyl cellulose (CMC) (BrataChem, Bandung, Indonesia), Bouin solution, distilled water (BrataChem, Bandung, Indonesia), mouse monoclonal IgG1 NF-kappaB p65 antibody (Santacruz Biotechnology Inc., K2320, Shanghai, China), β-actin antibody (Thermo Scientific MA5-15739, Singapore), anti-mouse antibody (Li-Cor C90408-07, PT. Elo Karsa Utama, Jakarta, Indonesia), bovine serum albumin (BSA Protein, Thermo Scientific, Singapore), sodium dodecyl sulfate (SDS) gel 15% (Merck Millipore-Sigma Aldrich, St. Louis, MI, USA), polyvinylidene difluoride (PVDF) membrane (Merck Millipore-Sigma Aldrich, St. Louis, MI, USA), phosphate buffer saline-tween 20 (PBST) 0.1%, protein ladder (Thermo Scientific, Singapore), running buffer, transfer buffer, lysis buffer.

### 4.2. Crab Shells

Fresh blue swimming crab shells were collected in a traditional market in Gunung Jati Cirebon, the northern part of West Java, with size ranges of 13–15 cm. The shells were boiled, washed meticulously to remove meat residue and dirt, and sun-dried for three days. The dried shells were ground to powder and sieved using a sieve mesh-60. The powder was put in sealed plastic pouches with silica gels until further processed.

### 4.3. Extraction of Chitin and Analysis of the Physicochemical Properties

The extraction of chitin was carried out following a previous study employing a microwave-assisted extraction method [8]. The blue swimming crab shell powder was dissolved in a mixture of 69.8 g of choline chloride (C_5_H_14_ClNO; molecular weight of 139.62) and 67.045 g of DL-malic acid (C_4_H_6_O_5_; molecular weight of 134.09) in 1 L of distilled water. The mixture was stirred at 80 °C until homogenous, then was put in a microwave (Rewez Multifunctional) at 700 watts for 9 min, and every minute it was taken out for three seconds to prevent overheating. Chitin, yielded as precipitate, was separated from the solvent by centrifugation (80-1 tabletop low-speed centrifuge), washed using distilled water, and the extraction was repeated 2×. This extraction technique yielded 35.43% *w*/*w* of yellowish-white chitin precipitate.

The physicochemical properties, in terms of the water content (gravimetric method), protein (Kjeldahl method), lipid (Soxhlet method), and carbohydrate (by difference method) in the extract were determined by following the Indonesian Standardization Agency (1992) Methods for Analyzing Food and Beverages (SNI 01-2891-1992) [42]. The levels of five toxic heavy metals (mercury at 253.6 nm, lead at 220.4 nm, chromium at 283.6 nm, cadmium at 226.5 nm, and arsenic at 188.9 nm) were also analyzed using an Inductively Coupled Plasma Optical Emission spectroscopy (Agilent Technologies 725). These works were done at the Central Laboratory, Directorate of Research and Community Engagement, Padjadjaran University, in October 2020.

The deacetylation degree (**DD**) of chitin extract was (1) determined by Fourier Transform-Infrared spectrometer, which is based on the absorbance (***A***) ratio of the amide band of the acetyl group at 1650 cm^−1^ and the OH (hydroxyl) group band at 3450 cm^−1^ by following the equation of [27,28,29]:

**DD (%) = 100 − 75.19 [*A*_1650_/*A*_3450_]**

The chitin-potassium bromide pellets were prepared by dispersing 1 mg of dry chitin extract powder in 200 mg of potassium bromide and were compressed under a vacuum for 10 min to obtain the potassium bromide discs. The discs were recorded in an FT-IR spectrometer (Nicolet^TM^ 380 FT-IR ThermoFisher Scientific) at a frequency range of 4000–500 cm^−1^.

The molecular weight (MW) of chitin was calculated by determining the viscosity of 1% chitin solution in acetic acid. The solution was stirred for 4 h, measured in an Ostwald viscometer, and the viscosity was calculated by following an equation of Mark–Houwink [43]:

**[*η*] = *KM*^a^ or Log [*η*] = Log *K* + a (Log *M*)**

where ***η*** = intrinsic viscosity (in centipoise, cP), ***K*** = 1.74 × 10^−5^ L/g and **a** (the slope), both of which are constants depending on the **DD** of chitin; and ***M*** = molecular weight.

### 4.4. Animals and Ethical Considerations

Forty-four male Wistar rats, 10–12 weeks, 190–210 g were used in this study. The number of animals used was calculated using the Federer Formula: (12 − 1) × (n − 1) ≥ 15, which resulted in n = 4 for each group, but only data from 3 animals in each group were included. No animals were found dead during the study (the duration of the study was 40 days). Rat handling and euthanasia procedures were performed as approved by the Research Ethics Committee, Padjadjaran University, Indonesia, recognized by the Forum of Ethics Review Committee in Asia & Western Pacific Region (approval document No. 541/UN6. KEP/EC/2020).

### 4.5. Animal Preparation and Treatment Groups

The in vivo experiment was carried out in the Pharmacology Laboratory, School of Pharmacy Muhammadiyah Cirebon, West Java. The design of the study strictly followed Essential 10 of the ARRIVE guidelines (Animal Research: Reporting of In Vivo Experiments) [44]: Forty-four male Wistar rats were randomly assigned into 11 groups (n = 4) and treated for 13 consecutive days. The rats were kept in animal cages (cage base area 148 cm^2^, cage height 20 cm) at 24–26 °C under a 12 h light, 12 h dark cycle, and were provided with standard pellet food (Citra Feed RatBio) 150 g/cage daily and water freely for 7 days. Animal health and behavior were observed daily. The cages were cleaned every 2 days:

- Group 1: positive control, treated with sucralfate (1080 mg/kg BW);
- Group 2: negative control or placebo, treated with CMC 1% suspension;
- Group 3: normal control, without any treatment;
- Group 4: treated with chitin extract (dose of 150 mg/kg BW);
- Group 5: treated with chitin extract (dose of 300 mg/kg BW);
- Group 6: treated with chitin extract (dose of 600 mg/kg BW);
- Group 7: treated with commercial chitin (dose of 150 mg/kg BW);
- Group 8: treated with commercial chitin (dose of 300 mg/kg BW);
- Group 9: treated with commercial chitin (dose of 600 mg/kg BW);
- Group 10: treated with crab shell powder (dose of 500 mg/kg BW);
- Group 11: treated with crab shell powder (dose of 1000 mg/kg BW).

On day 14 before the initiation of gastric ulcer, the rats were not given feed for 12 h to establish an empty stomach (the drink was still given freely).

### 4.6. Induction of Ulcer

**Figure 6:**
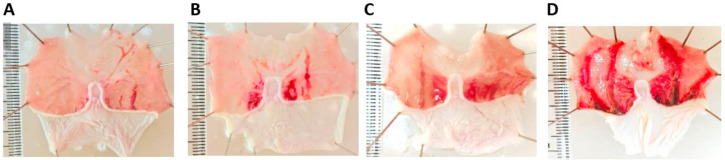
The rat’s stomach after ethanol-induced ulcerative at the time of (**A**) 30 min; (**B**) 1 h; (**C**) 1.5 h; (**D**) 2 h.

The initiation of ulcers in all rats, except the normal group, was performed by orally administering absolute ethanol at a dose of 5 mL/kg BW using an intragastric feeder, as delineated previously [45]. The optimization of the ethanol-ulcerative inducing time before the animal sacrifice was determined as follows using a total of 4 rats for each different time: 30 min; 1 h; 1.5 h; 2 h. None of the rats died prematurely prior to euthanasia. The death was verified with the absence of pupillary to direct and consensual light, pupils not equal or dilated, failure of the heart rate, and absence of respiratory efforts. The result is depicted in Figure 6. Based on this result, the inducing time of 1 h was chosen, because the severity of the hemorrhagic area is not as large compared to the inducing time of 1.5 h and 2 h.

### 4.7. Relative Organ Weight

The rats were anesthetized using ether inhalation and sacrificed, 1 h after ulcer induction by cervical dislocation, and the stomach of all groups was separated, cleansed with 0.9% sodium chloride in water, macroscopically examined, and weighed. The relative stomach weight was calculated as follows:

$$\%\;Relative\;stomach\;weight=\left(\frac{stomach\;weight}{body\;weight}\right)\times 100\%$$

### 4.8. Histopathological Examination of the Stomach

The clean stomach of all groups was sectioned at the major curvature for histological studies. The stomach was fixed in a 10% Bouin solution. The Bouin-fixed stomach sections were embedded in paraffin wax, subsequently sliced to 2–3 μm thickness, stained with hematoxylin with 15 min soaking time and eosin with 4 min soaking time at room temperature (H&E), and perceived for pathologic diversities using an Olympus CX33 microscope, and the ulcer area was measured using ImageJ software. Ulcer index (%) was calculated as follows:

% Ulcer index = [ulcer area/total area of stomach mucosa] × 100%

### 4.9. Effect of Chitin towards the Infiltration of Inflammatory Cells

The infiltration of inflammatory cells on the gastric mucosa was observed using a light microscope with a magnification of 400× and 1000× to count the number of normal cells, necrotic cells, and fat degeneration.

### 4.10. Effect of Chitin towards NF-kappaB p65 in Ethanol-Induced Gastric Ulcerative Wistar Rats

The rats’ 2–3 μm thickness of stomach tissue was homogenized in a lysis buffer. The protein samples were heat-denatured at 95 °C for 5 min and cooled to room temperature. Samples (10–15 μg/lane) were separated by sodium dodecyl sulfate-polyacrylamide gel electrophoresis (SDS-PAGE) at an initial voltage of 80 V, followed by gradually increasing the voltage from 80 V, 100 V, 120 V, to 150 V for 1 h, conveyed to a nitrocellulose membrane (GE Healthcare) for 1 h at 20–22 °C, and occluded for twelve hours at 4 °C in Tris-buffered saline with 0.1% Tween^®^ 20. Immunoblotting was performed using a mouse monoclonal NF-kappaB p65 antibody (Santacruz Biotechnology Inc., diluted 1:100 in bovine serum albumin 0.2%). Blots were stripped, cleansed with phosphate-buffered saline Tween20 0.1% detergent (PBST) three times, and re-probed using a secondary antibody (anti-mouse monoclonal antibody in bovine serum albumin 0.2%) as the internal control, incubated for 2 h, and washed with PBST 0.1% three times. The signals were developed using an enhanced chemiluminescence reagent (GE Healthcare), scanned using LI-COR Odyssey scanner, and the intensity of the band was measured using ImageJ-Win64 (https://imagej.nih.gov/ij/ accessed on 31 May 2021).

### 4.11. Statistical Analysis

Data were analyzed using IBM SPSS Statistics version 24.0. Normally distributed data were analyzed using the One-way ANOVA, further analysis was performed with a post hoc test using LSD, and other data were analyzed using Mann-Whitney. *p*-value < 0.05 indicates a statistical significance. The experiments were performed in triplicates.

## 5. Conclusions

Chitin was successfully extracted from the shells of blue swimming crabs (*Portunus pelagicus* Linn.) using a mixture of choline chloride and DL-malic acid as the solvent. The physicochemical properties of the chitin extract confirmed its good quality. Chitin extract could protect the gastric mucosal cells against ethanol-induced ulcerative damage. It seems that the gastroprotective activity of chitin also advances the immune system by decreasing the expression of NF-kappaB p65 in the rats’ stomachs, thereby preventing the protein from translocating to the nucleus. Ultimately, the inflammatory process will not take place. Taking everything into consideration, chitin extracted from the shells of blue swimming crabs might be able to prevent gastric ulcers via the inhibition of NF-kappaB p65, thus confirming the health values of these seafood wastes. However, further studies are needed to verify its anti-ulcerative properties.

## Figures and Tables

**Table 1:** Effect of different doses of chitin extract, commercial chitin, and crude shell powder on the % relative stomach weight and % ulcer index in ethanol-induced gastric ulceration rats.

Rat Group	Stomach Index (%) ± SD	Ulcer Index (%)
Positive control group	0.62 ± 0.03	0.17 ± 0.11 *
Negative control group	0.63 ± 0.09	14.55 ± 2.62 ^#^
Normal control group	0.57 ± 0.04	0.06 ± 0.06 *
Chitin extract 150 mg/kg BW	0.54 ± 0.04	4.79 ± 4.37 *
Chitin extract 300 mg/kg BW	0.51 ± 0.02	1.08 ± 0.77 *
Chitin extract 600 mg/kg BW	0.49 ± 0.03	1.60 ± 0.44 *
Commercial chitin 150 mg/kg BW	0.72 ± 0.14	2.94 ± 1.74 *
Commercial chitin 300 mg/kg BW	0.62 ± 0.16	1.32 ± 0.44 *
Commercial chitin 600 mg/kg BW	0.59 ± 0.05	4.80 ± 0.66 *
Crude shell powder 500 mg/kg BW	0.59 ± 0.03	1.50 ± 1.17 *
Crude shell powder 1000 mg/kg BW	0.60 ± 0.07	1.42 ± 0.77 *

Statistical analysis was done using IBM SPSS Statistics version 24.0 for Windows. All experiments were replicated 3×. Data are presented as mean ± SD. * Indicates a significant difference (*p* < 0.05) compared to the negative control group. ^#^ Indicates a significant difference (*p* < 0.05) compared to the normal control group.

**Table 2:** Effect of different doses of chitin extract, commercial chitin, and crude shell powder on the number of necrotic cells, normal cells, and % fat degeneration in ethanol-induced gastric ulceration rats.

Rat Group	Necrotic Cells	Normal Cells	Fat Degeneration
Positive control group	57	883	22
Negative control group	88	836	48
Normal control group	43	911	20
Chitin extract 150 mg/kg BW	47	888	20
Chitin extract 300 mg/kg BW	67	864	26
Chitin extract 600 mg/kg BW	63	861	24
Commercial chitin 150 mg/kg BW	62	879	23
Commercial chitin 300 mg/kg BW	55	880	21
Commercial chitin 600 mg/kg BW	58	882	19
Crude shell powder 500 mg/kg BW	67	851	25
Crude shell powder 1000 mg/kg BW	77	848	23

No statistical analysis was done. Counting and observation were done 1× for each group.

## Data Availability

Not applicable.
